# Immunogenic cell death-based prognostic model for predicting the response to immunotherapy and common therapy in lung adenocarcinoma

**DOI:** 10.1038/s41598-023-40592-w

**Published:** 2023-08-16

**Authors:** Xiang Zhou, Ran Xu, Tong Lu, Chenghao Wang, Xiaoyan Chang, Bo Peng, Zhiping Shen, Lingqi Yao, Kaiyu Wang, Chengyu Xu, Jiaxin Shi, Ren Zhang, Jiaying Zhao, Linyou Zhang

**Affiliations:** Department of Thoracic Surgery, The Second Affiliated Hospital of Harbin Medical University, Harbin, China

**Keywords:** Immune cell death, Cancer models, Lung cancer, Tumour immunology

## Abstract

Lung adenocarcinoma (LUAD) is a malignant tumor in the respiratory system. The efficacy of current treatment modalities varies greatly, and individualization is evident. Therefore, finding biomarkers for predicting treatment prognosis and providing reference and guidance for formulating treatment options is urgent. Cancer immunotherapy has made distinct progress in the past decades and has a significant effect on LUAD. Immunogenic Cell Death (ICD) can reshape the tumor’s immune microenvironment, contributing to immunotherapy. Thus, exploring ICD biomarkers to construct a prognostic model might help individualized treatments. We used a lung adenocarcinoma (LUAD) dataset to identify ICD-related differentially expressed genes (DEGs). Then, these DEGs were clustered and divided into subgroups. We also performed variance analysis in different dimensions. Further, we established and validated a prognostic model by LASSO Cox regression analysis. The risk score in this model was used to evaluate prognostic differences by survival analysis. The treatment prognosis of various therapies were also predicted. LUAD samples were divided into two subgroups. The ICD-high subgroup was related to an immune-hot phenotype more sensitive to immunotherapy. The prognostic model was constructed based on six ICD-related DEGs. We found that high-risk score patients responded better to immunotherapy. The ICD prognostic model was validated as a standalone factor to evaluate the ICD subtype of individual LUAD patients, which might contribute to more effective therapies.

## Introduction

Cancer is the second leading cause of death after heart disease, representing 21% of total deaths. Most cancer deaths are from lung cancer due to the lowest mid-term survival. Non-small cell lung cancer (NSCLC) (85% of patients) and small cell lung cancer (SCLC) (15% of patients) are the two main types of lung cancer. The World Health Organization (WHO) classifies NSCLC into three main types: adenocarcinoma, squamous cell carcinoma, and large cell. The most common NSCLC type is adenocarcinoma accounting for 40% of lung cancers^1,2^. Various treatments are applied to cure lung adenocarcinoma (LUAD) patients, including surgery, chemotherapy, molecular-targeted therapy, radiotherapy, and immunotherapy^3^. Chemotherapy and molecular-targeted therapy are two common treatment strategies for LUAD, and the first-line treatment for LUAD patients is platinum-based combination chemotherapy^4^. In the past decades, cancer treatment has shifted with further research from tumor cells to tumor microenvironments^5^. Immunotherapy is an effective method to treat LUAD and is affected by the tumor immune microenvironment (TME).

Immunogenic Cell Death (ICD) is a part of Regulated Cell Death (RCD)^6^ and is triggered by obligate intracellular pathogens, therapeutic oncolytic viruses, molecules with oncolytic potential, conventional chemotherapeutics, epigenetic modifiers, targeted anticancer agents, and physical interventions^7^. The TME can be reshaped via the emission of danger signals or damage-associated molecular patterns (DAMPs)^8,9^, including the cell surface exposure of calreticulin (CRT)^10^, and heat-shock proteins (HSP70)^11^, extracellular release of adenosine triphosphate (ATP), high-mobility group box-1 (HMGB1)^12^, type I IFNs^13^, and members of the IL-1 cytokine family^14^, resulting in antitumor immunity activation in immunocompetent hosts^15^. Pattern recognition receptors can recognize most DAMPs. Extracellular ATP and surface-exposed CRT act as a “find me” and “eat me” signal toward immune cells. Dendritic cells mature after exposure to these molecules and activate T cells to increase antitumor responses^16^, thereby reshaping the TME.

However, Immunotherapy significantly varies between individuals, and the prognosis is uneven. Hence, biomarkers are urgently needed to predict patient prognosis and treatment outcomes.

Herein, we aimed to construct a predictive tool based on biomarkers found in ICD-related genes to assist in the fastest and best treatment regimens for patients according to their circumstances to get better therapeutic efficacy. First, we screened ICD-related genes correlated to prognosis. Then, we constructed and validated an ICD-related gene classification based on these genes. Each patient was assigned a risk score to reflect the prognosis and give advice on treatment regimens using this prognostic model.

## Results

### DEGs differentiation and division of patients into two subtypes

**Figure 1:**
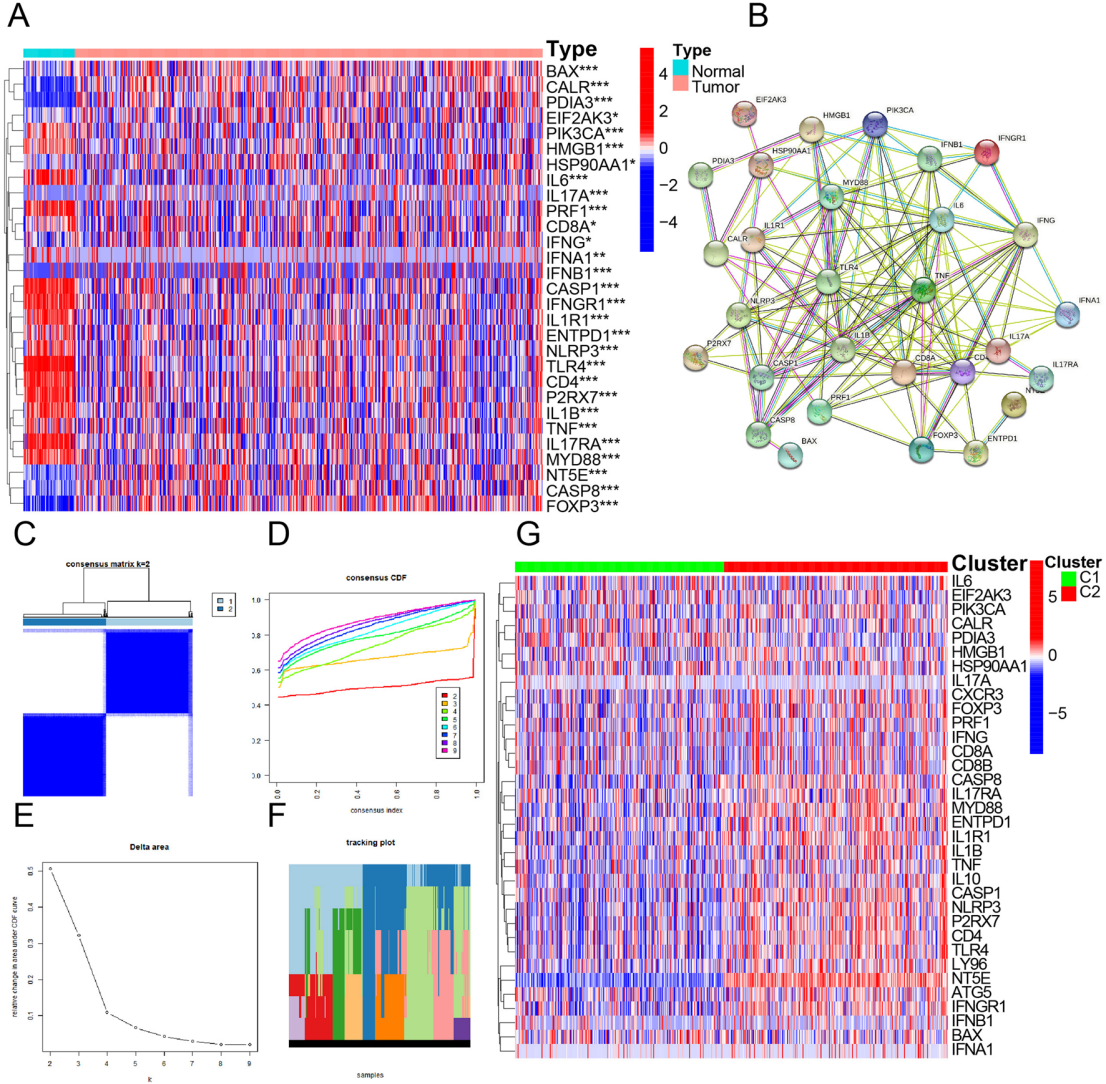
Identification of ICD-associated subtypes by consensus clustering. (**A**) Heatmap shows 34 ICD gene expression profiles among normal and LUAD samples in TCGA database. (**B**) Protein–protein interactions among the ICD-associated genes (**C**) Heatmap depicts consensus clustering solution (k = 2) for 34 genes in 539 LUAD samples (**D**,**E**) Delta area curve of consensus clustering indicates the relative change in area under the cumulative distribution function (CDF) curve for k = 2 to 9. (**F**) Tracking Plot of consensus clustering indicates the relative change of class that samples belongs to for k = 2 to 9. (**G**) Heatmap of 34 ICD-related gene expressions in different subtypes. Red represents high expression and blue represents low expression.

**Figure 2:**
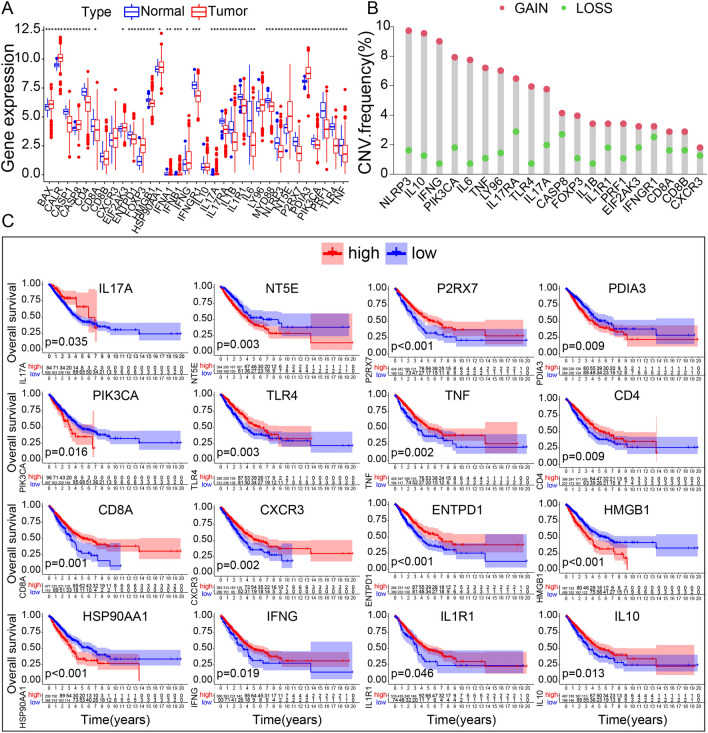
DEGs signature. (**A**) Box plot of genes expression in ICD-related DEGs. (**B**) CNV of 20 ICD-related DEGs. (**C**) Survival analysis of 16 ICD-related DEGs.

We filtered 34 ICD-related genes in 598 LUAD sets in 59 normal tissues and 539 LUAD samples from TCGA, according to an extensive literature summarized by Abhishek et al.^22^: ATG5, BAX CALR, CASP1, CASP8, CD4, CD8A, CD8B, CXCR3, EIF2AK3, ENTPD1, FOXP3, HMGB1, HSP90AA1, IFNA1, IFNB1, IFNG, IFNGR1, IL10, IL17A, IL17, RAIL1B, IL1R1, IL6, LY96, MYD88, NLRP3, NT5E, P2RX7, PDIA3, PIK3CA, PRF1, TLR4, and TNF. Then, the expression of these ICD-related genes was analyzed with the “pheatmap” R package to visualize the variance between tumor and normal samples. Twenty-nine genes were differentially expressed in tumor samples: 12 were upregulated (CD8A, BAX, EIF2AK3, HSP90AA1, CASP8, CALR, PDIA3, IFNG, FOXP3, NT5E, IL17A, and IFNB1), and 17 were downregulated (iIL6, TLR4, PRF1, IL1B, P2RX7, IFNGR1, CASP1, NLRP3, CD4, IL17RA, IL1R1, MYD88, IFNA1, TNF, ENTPD1, HMGB1, and PIK3CA) (Fig. 1A). These differentially expressed ICD-related genes between tumor and normal samples play a crucial role in the initiation and progression of tumors. The 29 ICD-related DEGs were used for PPI analysis via the STRING database (Fig. 1B). Then, we determined ICD-related clusters of LUAD by consensus clustering. LUAD samples were grouped into two subgroups that maximized consensus within clusters while minimizing ambiguity in cluster assignments and considering the feasibility of clinical prognosis analysis (Fig. 1C–F). LUAD samples were divided into two cohorts (C1 and C2), rendered in the landscape to present the DEGs (Fig. 1G). ICD-related genes were downregulated in C1, representing the ICD-low subgroup, while ICD-related genes were upregulated in C2, comprising the ICD-high subgroup. Meanwhile, most of DEGs shows differences in gene expression, CNV frequency and survival(Fig. 2A). Their gain copy levels are higher than loss copy levels(Fig. 2B). 16 DEGs have significant differences in survival analysis, among which the low expression of ENTPD1, IL17A, P2RX7, TLR4, TNF, CD4, CD8A, CXCR3, IFNG, IL1R1 and IL10 indicate worse outcome(Fig. 2C).

### Inter-groups differentiation and enrichment analyses

**Figure 3:**
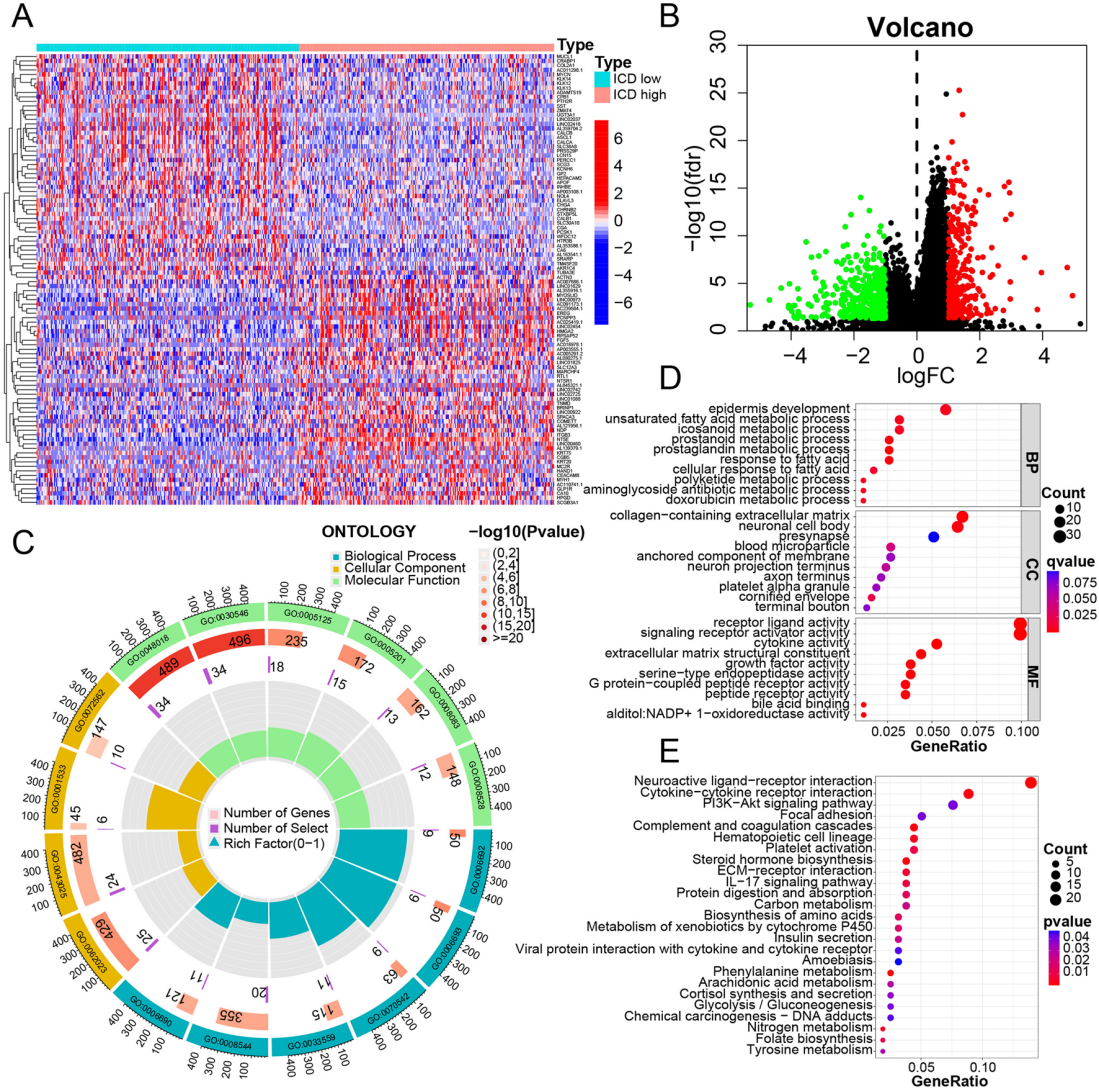
Identification of differentially expressed genes (DEGs) and underlying signal pathways in different subtypes. (**A**) Heatmap shows the DEG expression in different subgroups. (**B**) Volcano plot presents the distribution of DEGs quantified between ICD-high and ICD-low subtypes with threshold of |log2 Fold change|> 1 and *P* < 0.05 in TCGA cohort. (**C**) GO circle presents the signaling pathway enrichment analysis. (**D**,**E**) Dots plot presents the KEGG and GO signaling pathway enrichment analysis. The size of the dot represents gene count, and the color of the dot represents–log10 (*p* adjust-value).

**Figure 4:**
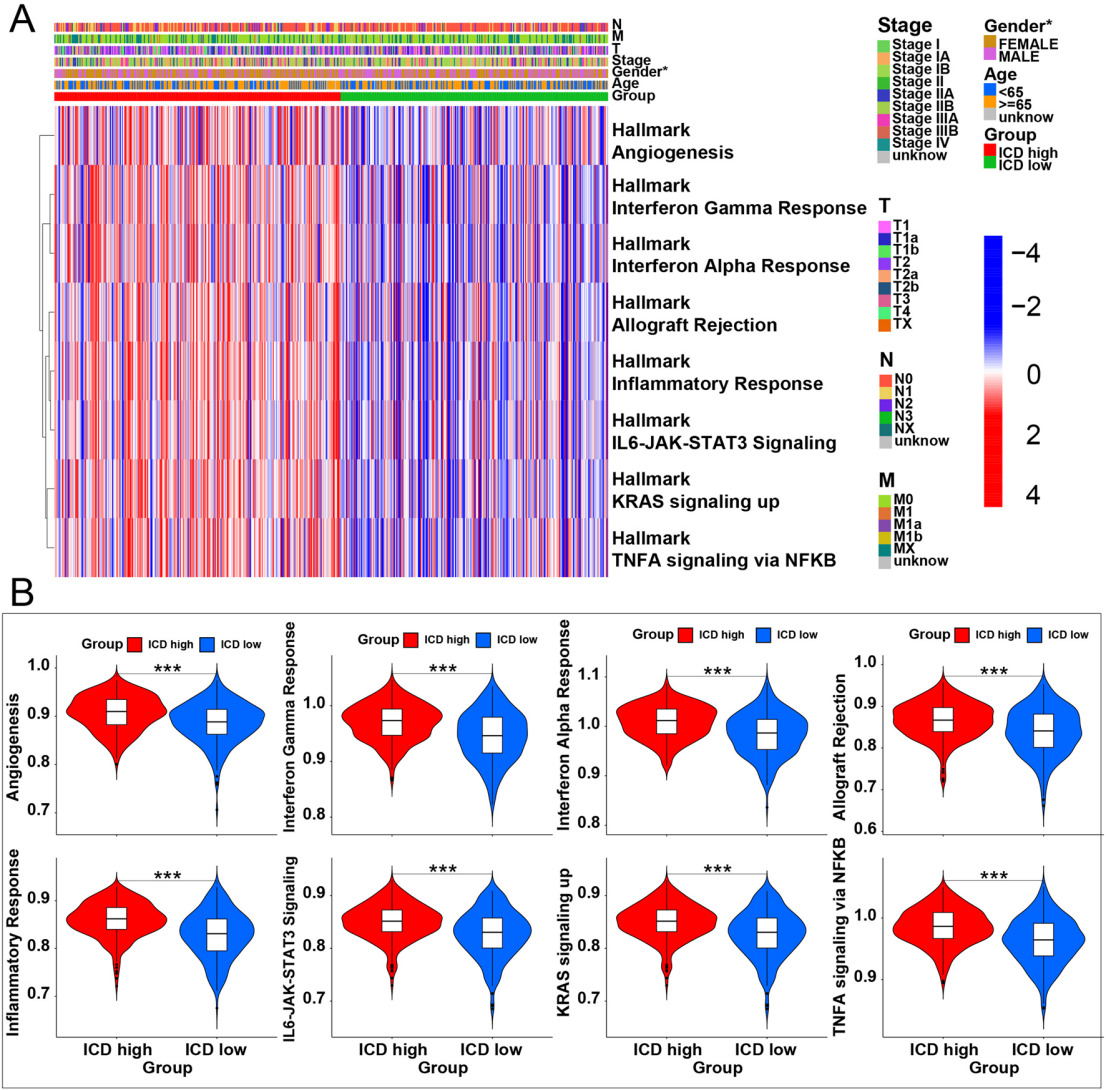
GSVA analysis. (**A**) Heatmap of GSVA enrichment analysis. (**B**) Violin plots show the differences in 8 GAVA enrichment signal pathways.

**Figure 5:**
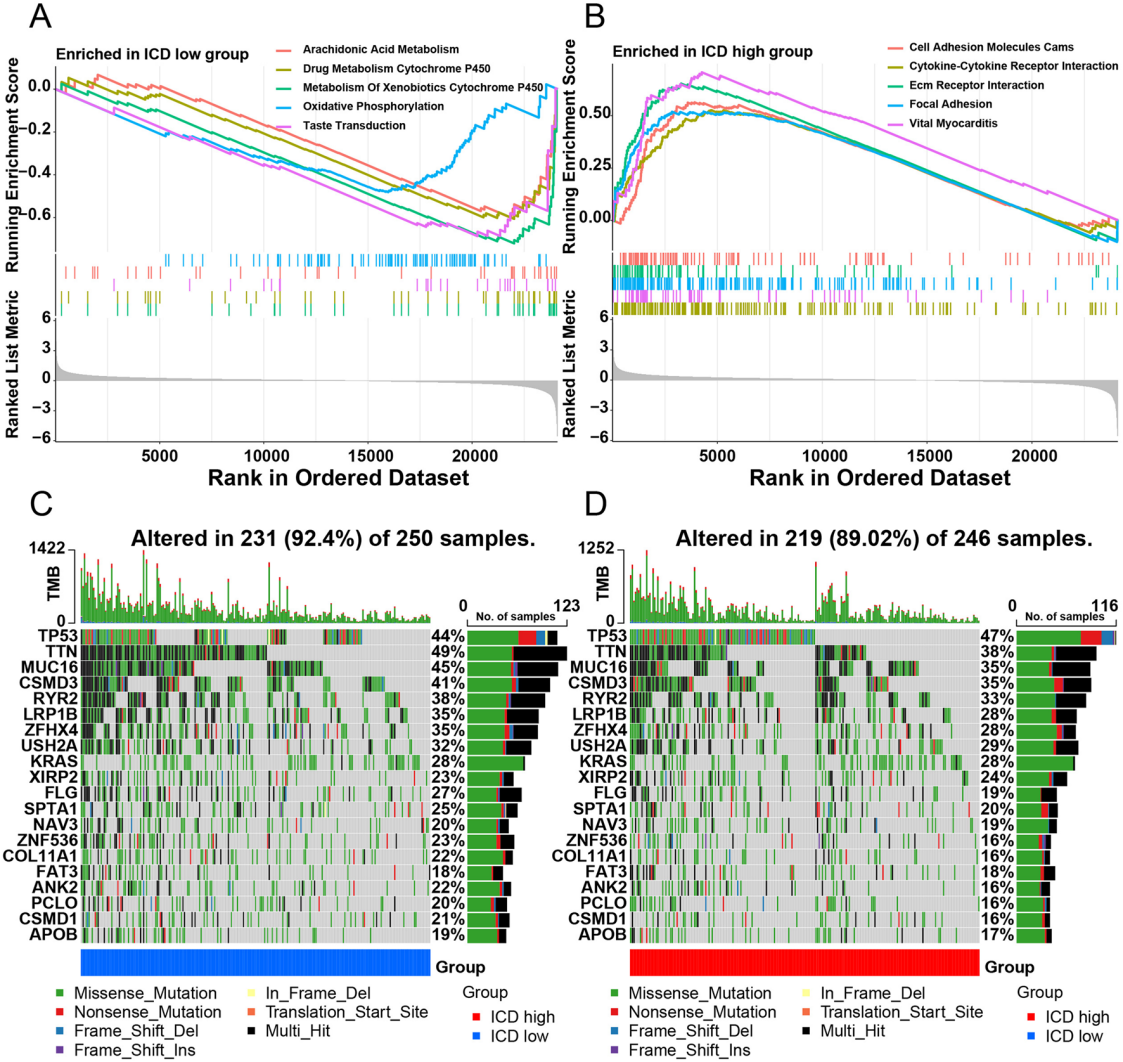
Comparison of GSEA signaling pathway and somatic mutations between different ICD subtypes. (**A**) GSEA analysis presents the signal pathway in ICD-low subtype. (**B)** GSEA analysis presents the signal pathway in ICD-high subtype. (**C**) Oncoplot visualization of the top twenty most frequently mutated genes in ICD-low subtype. (**D**) Oncoplot visualization of the top twenty most frequently mutated genes in ICD-high subtype.

All ICD-related genes were visualized in the landscape (Fig. 3A), where red cubes represent overexpression, while blue ones indicate underexpression. The volcano plot showed the gene distribution: red plot: upregulated; dark plot: unregulated; green plot: downregulated (Fig. 3B). The GO enrichment analysis comprises different classes: Biological Processes (BP), Cellular Components (CC), and Molecular Functions (MF) (Fig. 3C). This analysis revealed that DEGs were mainly enriched in BP related to immune function, such as receptor-ligand activity (GO:0048018); signaling receptor activator activity (GO:0030546) in the MF subgroup; and collagen − containing extracellular matrix (GO:0062023) in CC, which might contribute to immunotherapy. The same result can be seen in the dot plot representing gene ratios (Fig. 3D). Cytokine-cytokine receptor interaction was significantly enriched in the KEGG enrichment analysis. This pathway influences immune response processes such as adaptive inflammatory host defenses and cell death (Fig. 3E). The GSVA indicated that eight hallmark gene sets were differentially enriched between the two subgroups (Fig. 4A), all upregulated in the ICD-high subgroup (Fig. 4B). These pathways contribute to the response to Interferon-gamma, alpha interferon proteins, tumor necrosis factor, transplant rejection, inflammatory response, and KRAS^17^, indicating a higher level of tumor immune response in the ICD-high subgroup. The GSEA indicated that the cytokine-cytokine receptor interaction pathway was significantly enriched for ICD-related genes in the high-risk subgroup, which is also related to immune response (Fig. 5A,B). Therefore, the ICD-high subgroup was strongly related to immune pathways.

### Somatic mutation and TME

**Figure 6:**
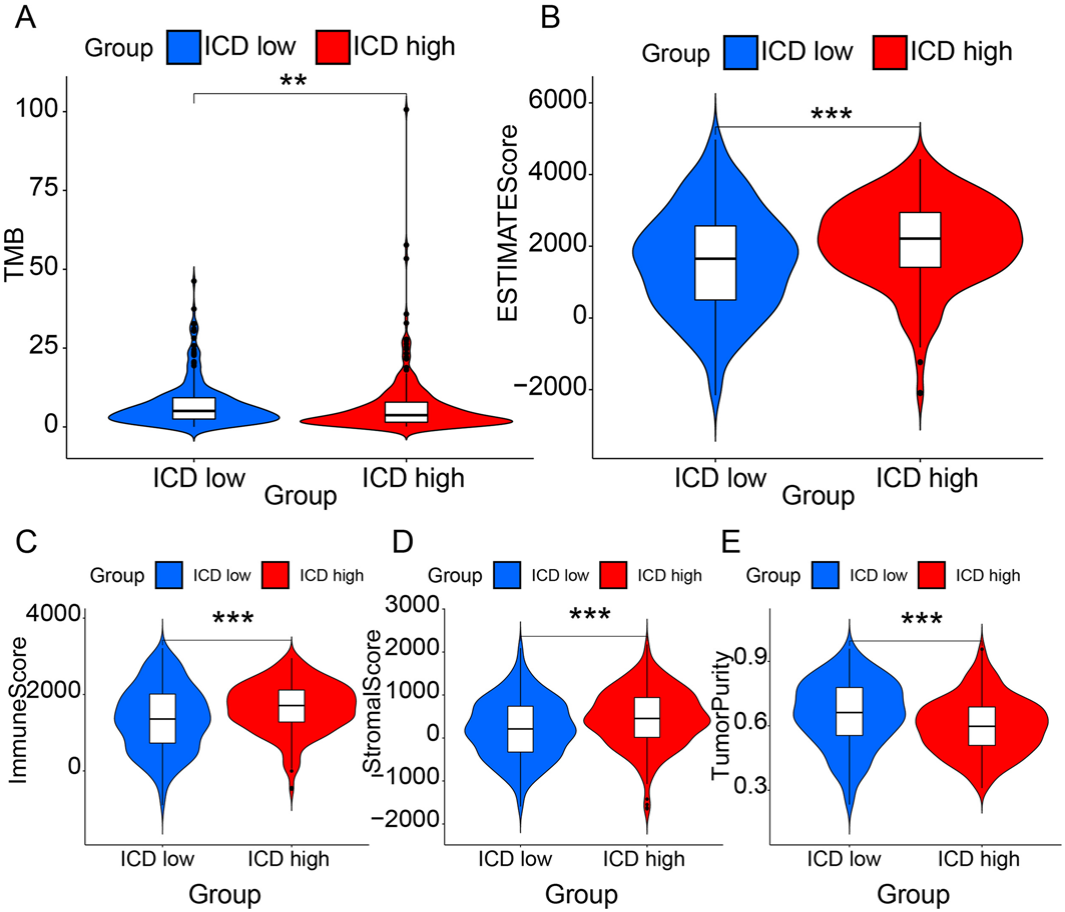
Tumor mutation burden difference and ingredient differences of tumor microenvironment between ICD-high and ICD-low subtypes. (**A**) Violin plots show *p* value, the median, and quartile estimations for tumor mutation burden between ICD-high and ICD-low subtypes. (**B**) Violin plots show *p* value, the median, and quartile estimations for estimate score between ICD-high and ICD-low subtypes. (**C**) Violin plots show *p* value, the median, and quartile estimations for immune score between ICD-high and ICD-low subtypes. (**D**) Violin plots show *p* value, the median, and quartile estimations for stromal score between ICD-high and ICD-low subtypes. (**E**) Violin plots show *p* value, the median, and quartile estimations for tumor purity between ICD-high and ICD-low subtypes.

**Figure 7:**
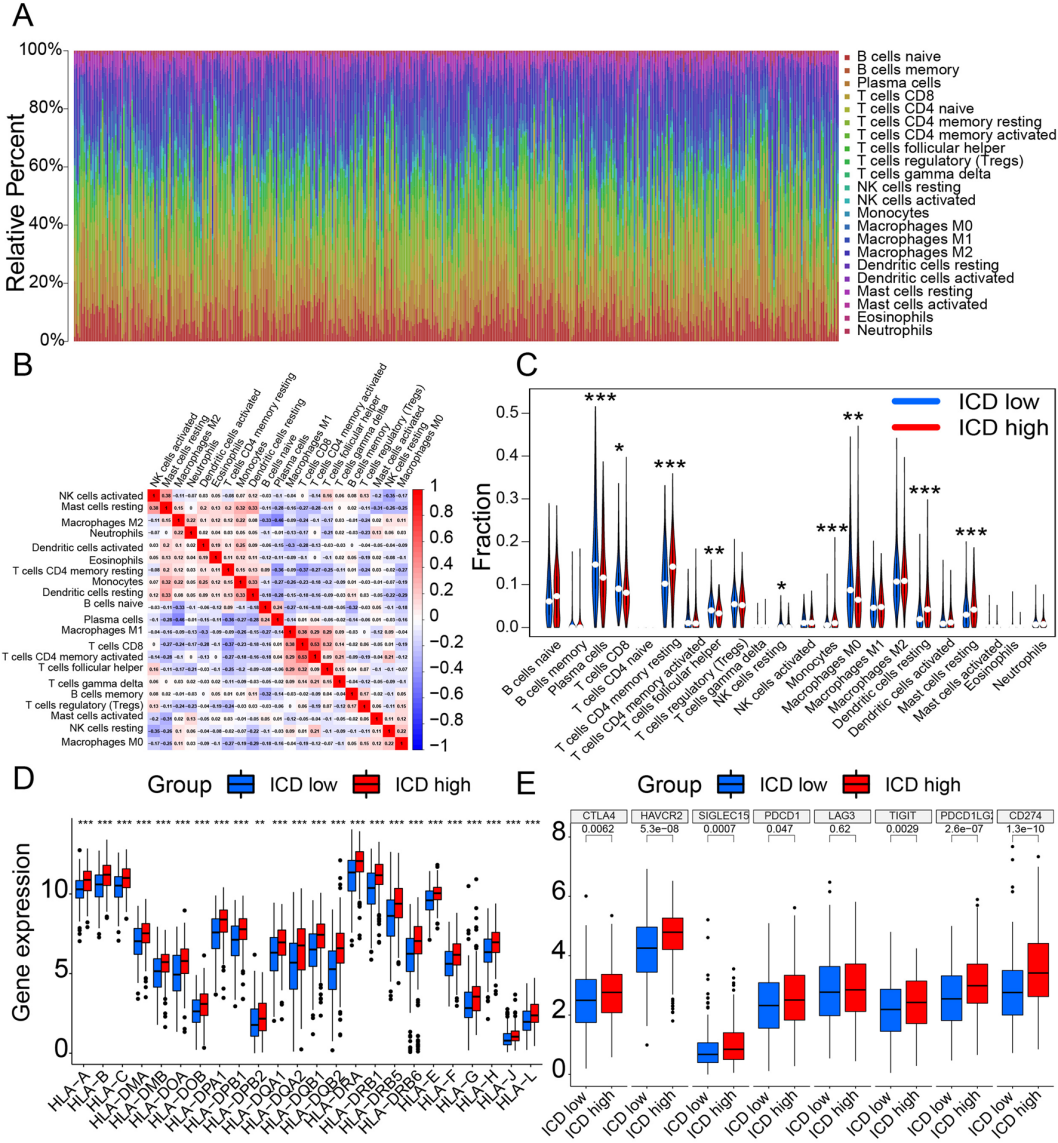
Immune landscape of ICD-high and ICD-low subtypes. (**A**) Relative proportion of immune infiltration in ICD-high and ICD-low subtypes. (**B**) Presentation of the correlation analysis of immune cells. (**C**) Violin plot visualizes significantly different immune cells between different subtypes. (**D**) Box plots present differential expression of HLA genes between ICD-high and ICD-low subtypes. (**E**) Box plots present differential expression of multiple immune checkpoints between ICD-high and ICD-low subtypes.

Further, we explored the distinct somatic mutation profiles between the two subgroups. To evaluate the somatic mutation differences between ICD-high and low subgroups, we drew the oncoplot for each subgroup (Fig. 5C,D). The frequency varied, as shown in the two charts. Despite TP53 and TTN that were high-frequency mutation genes, other genes, such as MUC16 and CSMD3, also differed. The ICD-high subgroup presented a lower mutation percentage than the ICD-low subgroup in different genes. The main mutation modes in the two subgroups are missense mutations and multi-hits. Further, we analyzed the tumor mutation burden (TMB) of the two subgroups. The ICD-low subgroup presented a statistically higher TMB than the ICD-high subgroup (Fig. 6A). Thus, upregulation of ICD-related genes might lower the TMB and inhibit tumor development. Then, we analyzed the immune microenvironment based on the ICD-related gene data. The four items differed between ICD-high and low subgroups. The estimate (Fig. 6B), immune (Fig. 6C), and stromal (Fig. 6D) scores were higher, and tumor purity (Fig. 6E) was lower in the ICD-high subgroup than in the ICD-low subgroup. Estimate scores are the sum of immune and stromal scores. These results demonstrated ICD’s ability to recruit immune infiltration. We obtained immune cell infiltration fractions using CIBERSORT to deconvolute samples from both subgroups. The content and relevance of 22 types of immune cells in ICD samples are presented (Fig. 7A). The correlation analysis of immune cells is shown (Fig. 7B). Thus, we contrasted ICD-high and low subgroups for each immune cell (Fig. 7C). CD4 memory resting T cells, monocytes, resting dendritic cells and resting mast cells were upregulated, while plasma cells, CD8T cells, follicular helper T cells, resting NK cells, and M0 macrophages were downregulated compared to the ICD-low subgroup with different *p* values (*). In the ICD-high subgroup, resting NK cells and M0 macrophages were downregulated, implying the improvement of the immune microenvironment. Furthermore, all human leukocyte antigen (HLA) genes listed below were overexpressed (Fig. 7D), and most checkpoints were similar (Fig. 7E) in the ICD-high subtype. Hence, the ICD-high subgroup was associated with the immune-hot phenotype, and the ICD-low subgroup was linked to the immune-cold phenotype.

### ICD prognostic model

**Figure 8:**
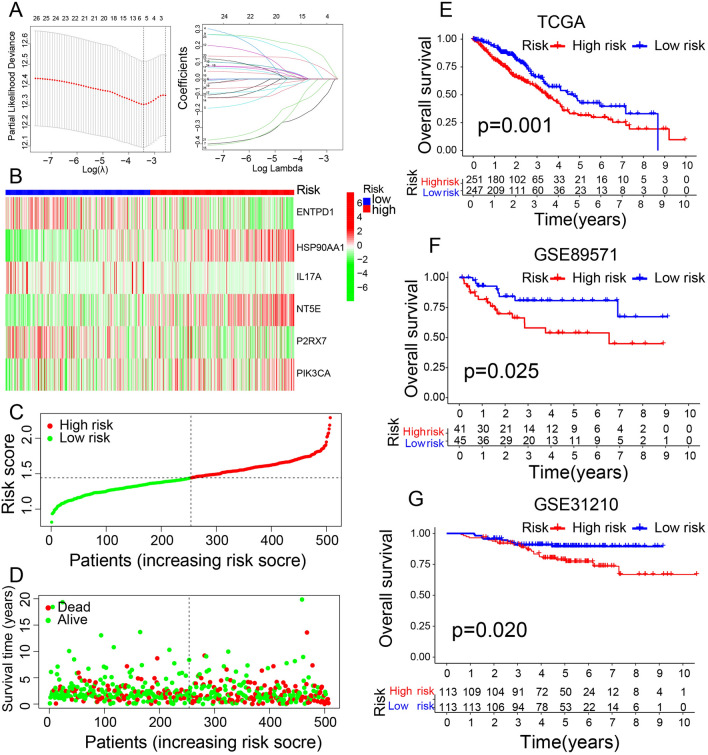
Construction and validation of ICD-related classification. (**A**) Lasso Cox analysis identified 6 genes most associated with OS in TCGA dataset. (**B**) Heatmaps of prognostic 6 gene signature in TCGA database. (**C**) Risk scores distribution of each patient in TCGA cohort. (**D**) Survival status of each patient in TCGA cohort. (**E**) Kaplan–Meier analyses demonstrate the prognostic significance of the risk model in TCGA cohort (**F**) Kaplan–Meier analyses demonstrate the prognostic significance of the risk model in GEO89571. (**G**) Kaplan–Meier analyses demonstrate the prognostic significance of the risk model in GEO31210.

**Figure 9:**
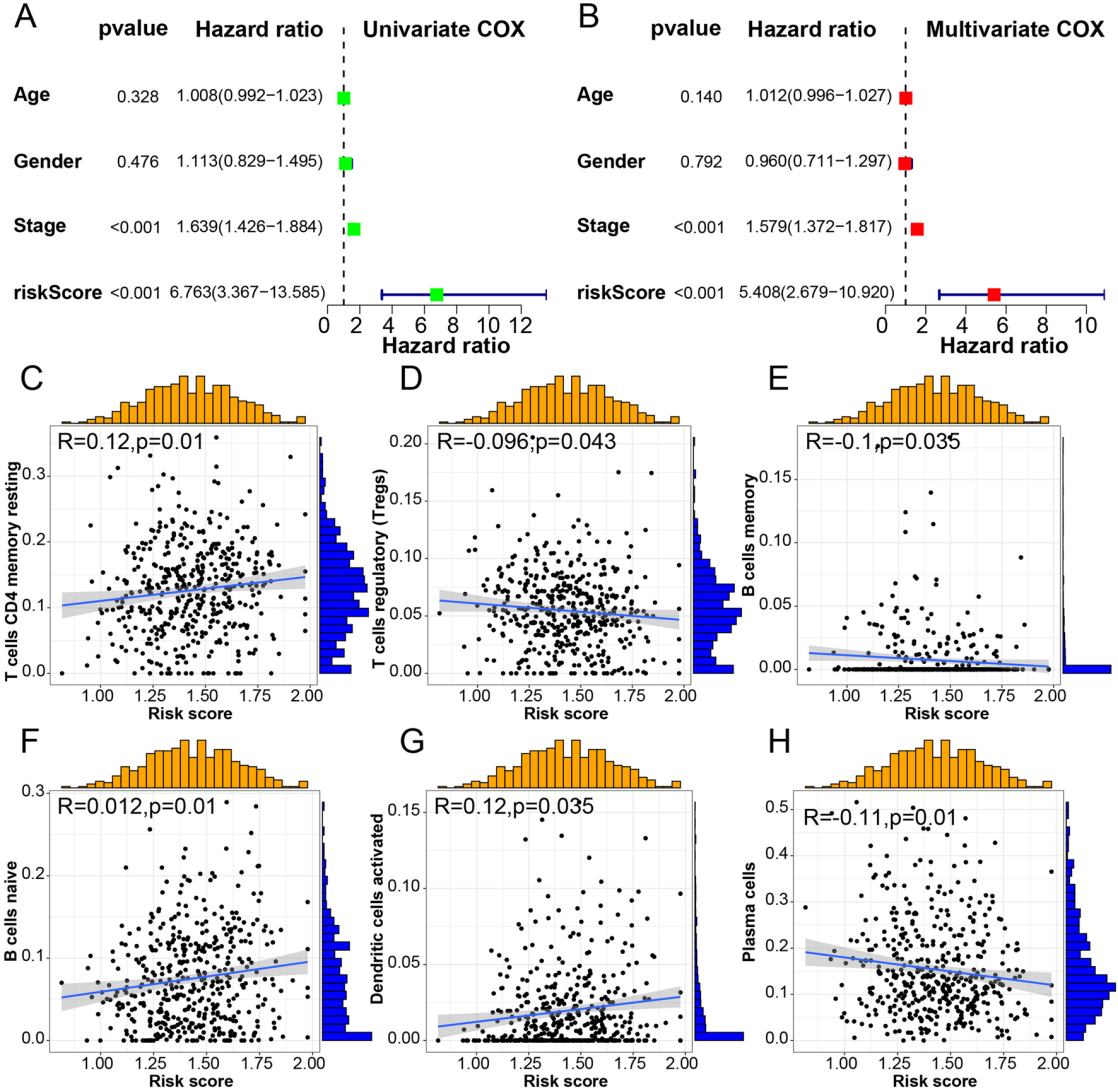
The association of ICD risk signature with tumor microenvironment. (**A**,**B**) Univariate and multivariate Cox analyses evaluate the independent prognostic value of ICD risk signature in LUAD patients. (**C**–**H**) Scatter plots show the correlation of risk score with the infiltration of T cells CD4 memory resting, Tregs, B cells memory, B cells naïve, Dendritic cells activated and Plasma cells.

Six ICD-related genes were selected from 16 DEGs previously mentioned for the classification model construction by LASSO regression analysis (Fig. 8A). They were associated with the patient survival (*p* < 0.05): ENTPD1, HSP90AA1, IL17A, P2RX7, PIK3CA, and NT5E. Each of them has a coefficient. Thus, we developed the classification based on the algorithm : Risk score = (0.1671) * HSP90AA1 + (0.0860) * NT5E + (−0.1682) *ENTPD1 + (0.0107) *IL17A + (0.0830) * P2RX7 + (0.0613) * PIK3CA. Each sample received a Risk Score. The landscape shows the individual risk score of the six ICD-related genes in increasing total Risk Score order (Fig. 8B). ENTPD1, IL17A, and P2RX7 were downregulated, while HSP90AA1, PIK3CA, and NT5E were upregulated, indicating that ENTPD1, IL17A, and P2RX7 might negatively affect tumor development and result in better outcome. We divided them into high- and low-risk subgroups (Fig. 8C). Moreover, the survival distribution indicated more deaths as the risk score increased (Fig. 8D). Then, we applied the Risk Score for samples in TCGA LUAD (Fig. 8E) and two cohorts of GEO LUAD (GSE89571, GSE31210) (Fig. 8F,G). In TCGA, a high-risk score was related to poor overall survival (OS), further confirmed in the GEO cohorts. This corresponds to the DEGs survival analysis. To verify the independent prognostic value of this classification, we used univariate and multivariate Cox regression analyses, which indicated the independent prognostic value of the ICD risk signature (*p* < 0.05). The univariate Cox regression analysis showed that a high ICD risk score was significantly associated with poor OS (Fig. 9A). The multivariate Cox regression analysis showed that the ICD risk score was an independent prognostic factor for LUAD patients (Fig. 9B).

### TME and therapy analysis

**Figure 10:**
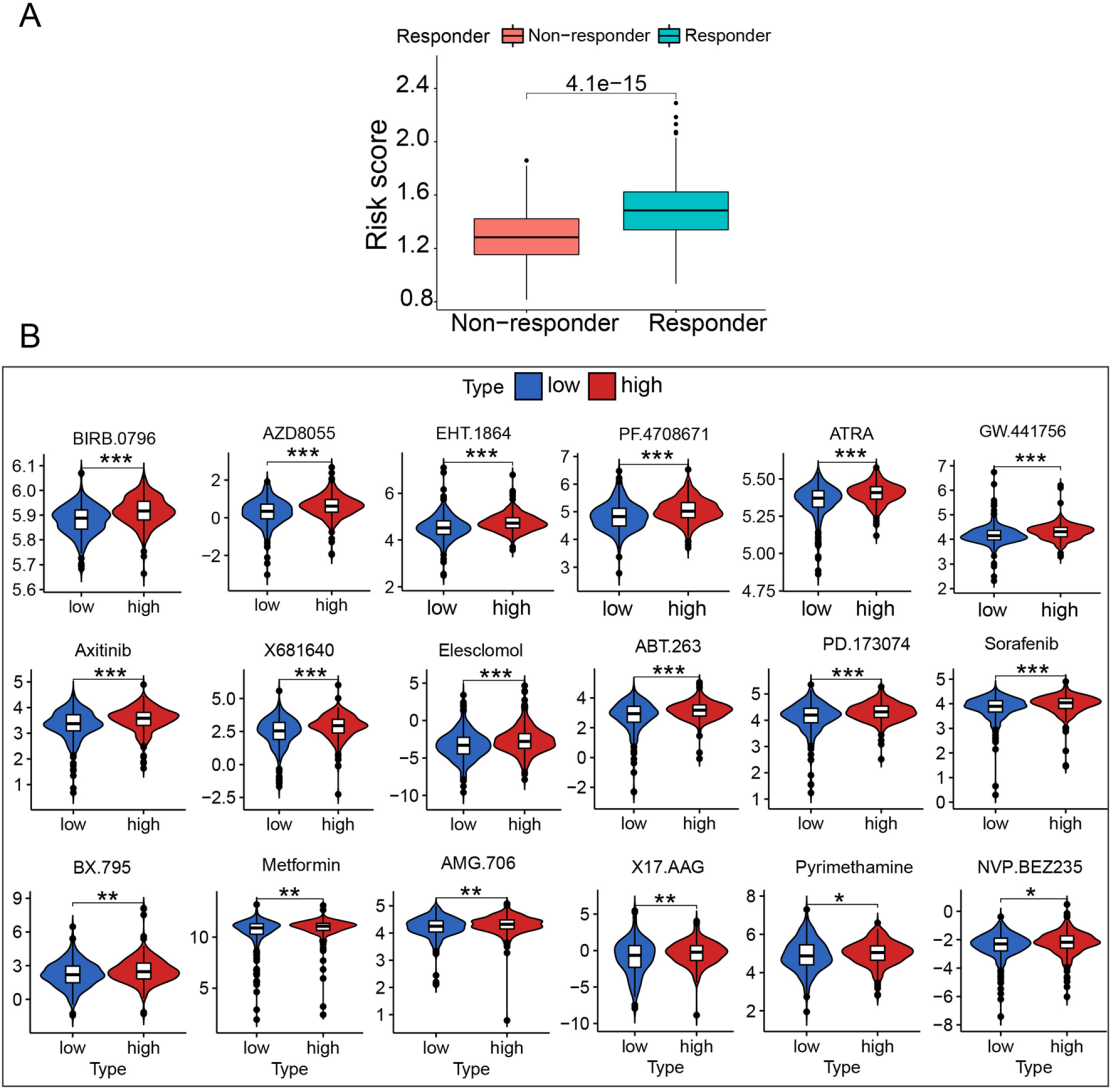
immunotherapy response and predicted treatment efficacy of common chemotherapy drugs. (**A**) Box plot presents the association of ICD risk score with immunotherapy response. (**B**) Predicted treatment efficacy of 18 common chemotherapy drugs.

ICD plays a vital biological role in anti-cancer immunological responses. Thus, it has a tight connection with the TME. In the immune correlation analysis, Plasma cells, T regulatory cells (Tregs), CD4 memory resting T cells, B cells memory, B cells naïve, endritic cells activated varied with the Risk Score (*p* < 0.05)(Fig. 9C–H). As the Risk Score increased, the concentration of Tregs decreased, and CD4 memory resting T cells increased (Fig. 9C,D). TIDE was used to evaluate the prognostic value of the Risk Score in immunotherapy based on a comprehensive analysis of the tumor expression spectrum to predict the efficacy of ICD therapy (Fig. 10A). The high-risk score group responded better to immunotherapy. Then, we used the drug information in the GDSC database to evaluate the IC_50_ of common chemotherapy drugs(Fig. 10B). We found lower IC_50_ for 18 chemotherapy drugs in the low-risk score subgroup than in the high-risk score subgroup: BIRB.0796, AZD8055, EHT.1864, PF.4708671, ATRA, GW.441756, Axitinib, X681640, Elesclomol, ABT.263, PD.173074, Sorafenib, BX.795, Metformin, AMG.706, X17.AAG, Pyrimethamine, and NVP.BEZ235. These drugs were statistically sensitive to low-risk score LUAD patients, providing basic selection advice of chemotherapy drugs alone or combined with immunotherapy in clinical practice.

### Single-cell analysis and experimental validation

**Figure 11:**
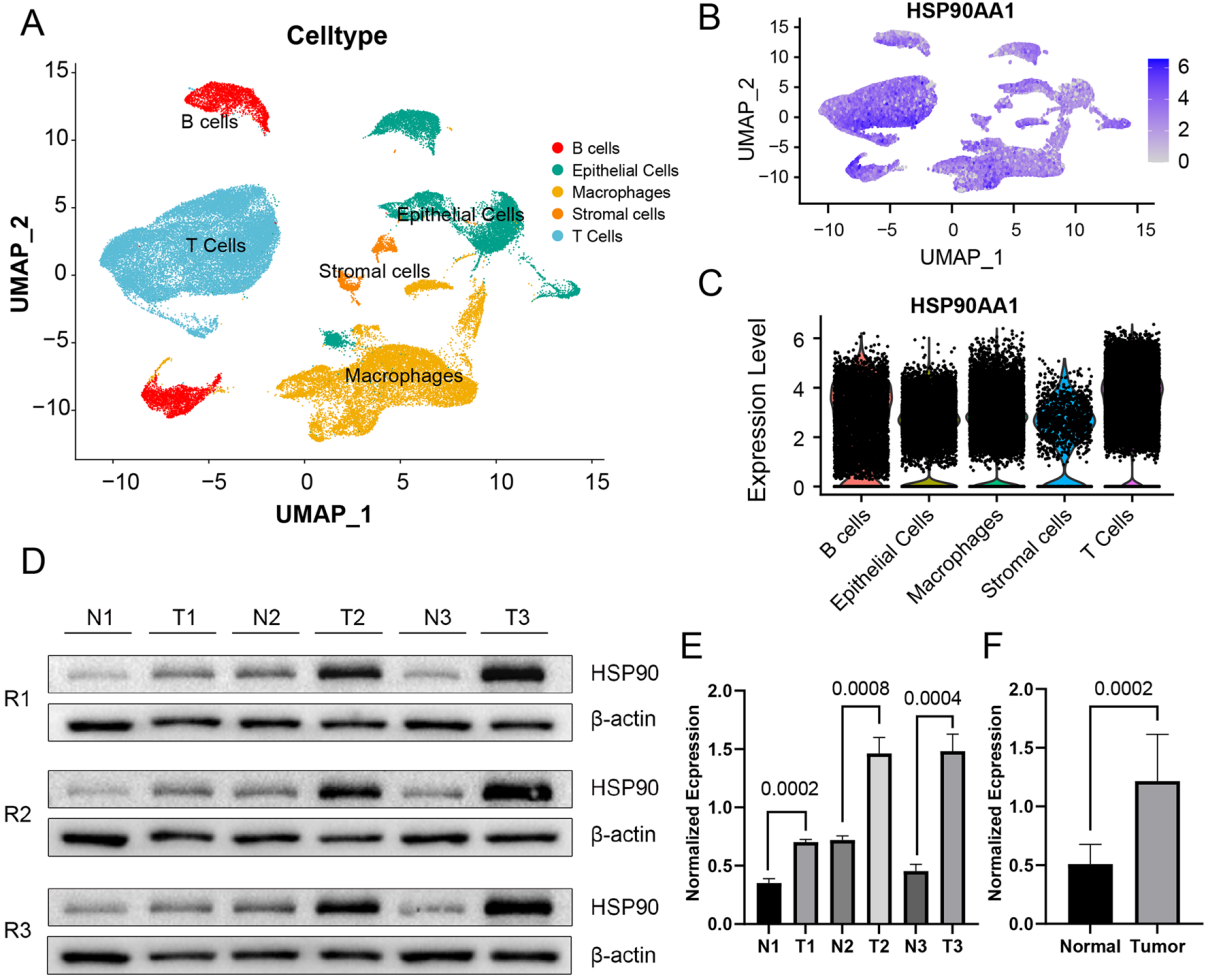
Single-cell analysis and Western blot validation. (**A**) Cellular distribution of 53504 cells clustered into 5 unique subsets among 10 lung adenocarcinoma tissue samples. (**B**) FeaturePlot depicting the distribution of HSP90AA1. (**C**) vlnPlot showing the expression levels of HSP90 in different cell subsets. (**D**) Western blot analysis of 3 paired LUAD and adjacent normal tissues with triplicate repeats. (**E**) Normalized expression differences of 3 different paired tissues (N represents normal,T represents tumor; N1 vs. T1 *p* value = 0.0002, N2 vs. T2 *p* value = 0.0008, N3 vs. T3 *p* value = 0.0004). (**F**) Normalized expression difference between normal and tumor tissues (*p* value = 0.0002).

With the intention of deeper investigation for these six ICD-related genes, we performed scRNAseq analysis with 10 LUAD samsamples. After the quality control process(Figure S1A),we manually identified 53504 cells which were distinguished into epithelial cells, stromal cells, macrophages, B cells and T Cells(Fig. 11A). The cell biomarkers was used for this process(Fig. S1B). In order to figure out the expression details of the six ICD-related genes in cell level, FeaturePlot and vlnPlot were performed(Figure S1C,D). We found that HSP90AA1 has higher expression than other five genes in all five types of cells(Fig. 11B,C). We conducted Western blot analysis to confirm the differential expression of HSP90AA1 between tumor and normal tissues. Tumor tissues and adjacent non-tumor tissues were obtained from three histologically confirmed lung adenocarcinoma patients, and the Western blot experiments were performed with triplicate repeats for each sample(Fig. 11D). The findings demonstrated a significant increase in the expression of the HSP90AA1 protein product in lung adenocarcinoma tissues compared to the levels observed in normal tissues, respectively(Fig. 11E,F).

## Discussion

Lung Adenocarcinoma (LUAD) accounts for 40% of lung cancer cases^2^. Surgery, radiotherapy, chemotherapy, targeted therapy, and immunotherapy are the mainstream treatment methods used for LUAD^3^. The efficacy of immunotherapy combined with classical cancer therapy, such as chemotherapy and targeted therapy, is remarkable^18–21^. However, there are significant differences between individuals in response to treatments. Hence, biomarkers are crucial to determine personalized treatment regimens for each patient to gain the best therapeutic effect. Thus, constructing an ICD-related gene classification to determine LUAD sensitivity to immunotherapy seems vital.

ICD is a unique RCD that works via DAMPs and danger signal emissions, triggering full antigen-specific adaptive immunological responses^7,22,23^. Dying cancer cells can activate this pathway, contributing to immunotherapy. Thus, ICD can be applied in cancer immunotherapy. Currently, no study has analyzed ICD-related prognostic genes in LUAD. Hence, constructing a prognostic model based on ICD-related prognostic genes to determine a risk score to predict a patient’s prognosis and treatment is of great interest.

Therefore, in the present study, we analyzed ICD-related biomarkers in LUAD and constructed an ICD prognostic model, which might be an easy and reliable tool for deciding treatment regimens (immunotherapy, other novel therapy, or combinations), leading to better outcomes.

Furthermore, we explored the genes and clinical data of 539 LUAD and 59 normal samples from TCGA and found 34 ICD-related DEGs. Then, we grouped LUAD samples into ICD-high and ICD-low subgroups based on ICD-related gene expression. These 34 ICD-related genes were previously summarized by Abhishek et al.^22^. We also used consensus clustering and selected k = 2, considering clinical factors and statistical parameters. The GO enrichment analysis indicated that most ICD-related genes were enriched for BP and CC. ICD-related genes affected receptor ligand activity, signaling receptor activator activity, and collagen − containing extracellular matrix. These pathways mainly affect the immune system. The GSVA showed that eight pathways were upregulated in the ICD-high subgroup, indicating a higher immune response level. These results highlighted the function of ICD-related genes. Cytokine-cytokine receptor interaction was also significant in the KEGG enrichment and GSEA. Moreover, the TMB was higher in the ICD-low subgroup, suggesting that ICD-related genes contributed to lower TMB. In the immune infiltration analysis, CD8 T cells presented a distinctly higher expression in the ICD-low subgroup. These results indicated the same outcome for TMB. The immune infiltration analysis also showed a higher infiltration in the ICD-high group, showing a lower tumor purity and higher stromal component in the TME analysis and the biological role of ICD. Therefore, the ICD-high group was related to an immune-hot phenotype and the ICD-low group to an immune-cold phenotype.

Finally, 6 of the 34 ICD-related DEGs were significantly associated with the prognosis of LUAD patients: ENTPD1^24^, HSP90AA1^25^, IL17A^26^, P2RX7^27^, PIK3CA^28^, and NT5E^29^. HSP90AA1 is exposed to the cell surface and can be reduced by DAMPs^11^. These six genes were used to construct the ICD classification resulting in two cohorts: high- and low-risk subgroups. The survival analysis for TCGA and GEO samples supported the grouping efficiency using this classification. Furthermore, the high-risk score subgroup presented a poor OS in TCGA samples, which was confirmed in the GEO cohort. As the risk score rise, more deaths were detected in samples. The different components of immune cells were also positively or negatively correlated with the risk signature. The univariate Cox analysis indicated that tumor stage and risk score statistically influenced prognostic factors. Multivariate Cox analysis demonstrated that the risk score was an independent factor in predicting the clinical and immunotherapy outcomes for LUAD. High-risk score patients had a favorable response to immunotherapy and might have better therapeutic effects than low-risk score patients. Therefore, a greater use of immunotherapy in high-risk scores might help improve the OS, which will be the subject of further in-depth clinical studies. Next, we analyzed the sensitivity to chemotherapy drugs widely used in clinical practice between two cohorts. The IC_50_ of 18 chemotherapy drugs was significantly lower in the low-risk score group than in the high-risk score group, indicating higher sensitivity in low-risk score LUAD patients. Thus, these drugs might be effective in LUAD therapy, but more clinical evidence is required to corroborate it.

We further performed single-cell analysis and observed that HSP90AA1 (Heat Shock Protein 90 Alpha Family Class A Member 1) exhibits significant and widespread expression across multiple cell types. It is a protein coding gene located in 14q32.31. The protein encoded by HSP90AA1 is a molecular chaperone protein that plays a key role in facilitating the folding, stabilization, and functional activity of specific client proteins^30^. In cancer cells, HSP90AA1 encoded protein can form complexes with various tumor-associated proteins and participate in regulating their stability and activity. Elevated expression of HSP90AA1 encoded protein is closely associated with the growth, proliferation, survival, and invasive capabilities of tumor cells. It can interact with certain oncogenic proteins, such as kinases, transcription factors, and receptors, forming complexes that protect them from degradation by the protein degradation system. For other types of cells in lung adenocarcinoma tissues, the expression of HSP90AA1 elevated due to the complexity of tumor tissue and alterations in the tumor microenvironment. Thus, HSP90AA1 increases the potential for our ICD-related progonstic model to generalize well to different adenocarcinoma samples. By validating the expression levels of this gene through wet lab experiments, it becomes possible to further evaluate the model's generalization ability and prediction accuracy. As a result, Western Blot experiment revealed its evaluated expression in adenocarcinoma tissues. This further confirmed the generalization ability and prediction accuracy of our model. However, only the western blot experiment for validation seems not enough. We are aimed to perform further wet-lab experiments to support our findings.

In conclusion, we constructed a prognostic model based on 6 ICD-related genes. They all influence the survival outcome. HSP90AA1,NT5E, PIK3CA have the negative influences while ENTPD1, IL17A, P2RX7 have the positive influences. Our current results demonstrated that the ICD-based classification was an independent prognostic value to provide reliable guidance for treating LUAD patients, especially immunotherapy, contributing to individualized treatment. These findings might help in the precise treatment and fine management of LUAD patients, reducing the overall mortality of lung cancer patients based on the rational use of anti-cancer drugs.

## Materials and methods

### Datasets

The RNA-seq data of 598 LUAD patients (59 normal tissues and 539 LUAD samples) and clinical phenotype data (survival status, grade, gender, and age) of 522 LUAD patients were retrieved from The Cancer Genome Atlas (TCGA) (https://portal.gdc.cancer.gov/) as the training cohort. TCGA-LUAD simple nucleotide variation (SNV) (Masked Somatic Mutation proceed by VarScan) and copy number variation (CNV) (Masked Copy Number Segment) data were retrieved from TCGA GDC. Transcriptome and survival data of 96 patients were retrieved from the Gene Expression Omnibus (GEO) as the independent validation cohort (accession number: GSE68571; https://www.ncbi.nlm.nih.gov/geo/query/acc.cgi?acc=GSE68571). The “R” language (v. 4.2.1) and STRING database [functional protein association networks (string-db.org)] were used for analysis.

### Identification of differentially expressed genes (DEGs) and clustering

ICD-related DEGs between LUAD and normal tissues were determined using the “limma” R package^31^ with absolute values of log fold change (FC) > 1 and false discovery rate (FDR) adjusted-*p* < 0.05. Protein–protein interaction (PPI) network analysis was conducted using the STRING database [STRING: functional protein association networks (string-db.org)] to determine protein interactions and reveal the connections between these ICD-related genes. The “ConcensusClusterPlus” R package^32^ was used to identify ICD subtypes by unsupervised consensus clustering with k ranging from 2 to 10. To guarantee clustering stability, we repeated the process 1000 times. The optimal cluster number comprised the two maximized consensus within clusters while minimizing ambiguity in cluster assignments considering the feasibility of clinical prognosis analysis. The cluster landscape was represented using the “pheatmap” R package^33^. The ICD-DEGs landscape was also visualized with this package.

### DEGs signature and enrichment analysis

We analysed DEGs differences in gene expression, copy number variationa(CNV) frequency and survival. Gene Ontology (GO)^34^ and Kyoto Encyclopedia of Genes and Genomes (KEGG) enrichment analyses^35–38^ were used to evaluate differences between ICD-high and ICD-low subgroups regarding signaling pathways and biological processes with q- and *p* value thresholds of  < 0.05. We applied Gene Set Variation Analysis (GSVA) with the “GSVA” R package^29^. Based on expression profiles, Gene Set Enrichment Analysis (GSEA)^39^ was performed by the “clusterProfiler” R package with a curated gene set retrieved from MsigDB. An adjusted-*p* < 0.05 was considered statistically significant for the phenotype analysis.

### Somatic mutation analysis

Somatic mutations are linked to tumor cell sensitivity and anti-chemotherapy mechanisms. These mutations also affect antitumor drug response prediction^40^. ICD-related somatic mutation data in LUAD patients were analyzed using the “maftools” R package^41^ and visualized using “oncoplot”.

### Tumor microenvironment (TME) and immune infiltration analysis

We analyzed the TME with the “estimate” R package. The relative content of immune cells in each LUAD sample was analyzed using the “CIBERSORT” R package^42^. Subsequently, immune cell differences in ICD-high and low groups were evaluated. Moreover, the expression of HLA and immune checkpoint genes were compared between ICD-high and low subgroups.

### Prognostic model construction and validation

Gene expression data combined with clinical outcomes for each sample in TCGA and GEO datasets were used to develop and validate an ICD-based prognostic model. The model was constructed with LASSO Cox regression analysis. With the available Risk Score from the model, its prognostic value for LUAD was further determined via Kapla-Maier (KM) analysis. We also performed univariate and multivariate Cox regression analyses to confirm the independent prognosis value of the Risk Score.

### ICD therapy

We analyzed the immune relevance and response to immunotherapy based on the Risk Score obtained from the prognostic classification model. For patients insensitive to immunotherapy, we explored their sensitivity to common chemotherapy drugs. Tumor immune dysfunction and exclusion (TIDE) (http://tide.dfci.harvard.edu/) analysis was conducted to evaluate the immunotherapy response. TIDE is an analytic technique that predicts immunotherapy response using two major tumor immune evasion mechanisms: T cell dysfunction and T cell infiltration inhibited in tumors with low CTL levels^43^.

### Single-cell analysis

We performed Single-cell analysis with single-cell RNA sequencing (scRNA-seq) data retrived from Code Ocean. The R package “Seurat”^44^was utilized to preprocess scRNA-seq data of 10 LUAD samples. After normalization by the “NormalizeData” function with the normalization method set as “LogNormalize”, we then converted the normalized data into a Seurat Object. Samples with gene counts below 200 or above 3000, as well as those with a ribosomal RNA proportion exceeding 20% were filtered out to ensure the quality control. We identified the top 3000 genes with the highest variability with the “FindVariableFeatures” function in “seurat” and then reduced the dimensionality of the scRNA-seq data using principal component analysis (PCA). JackStraw analysis identified significant principal components (PCs). The first 15 PCs were utilized for cell clustering analysis according to the proportion of variance explained. Cells were visualized using uniform manifold approximation and projection (UMAP) dimensionality reduction techniques for cell classification. We identified Marker genes using adjusted *p* value < 0.01 and |log2 (fold change)|> 1 as threshold values. A manual annotation, as described in the study by Maynard et al. was used to identify and classify different cell types in scRNA-seq data. Finally, visualization of the cellular distribution for independent prognostic genes in the scRNA-seq dataset was performed with FeaturePlot and vlnPlot functions in “Seurat” package.

### Western blot

All specimens were obtained from the Department of Thoracic Surgery, The Second Affiliated Hospital of Harbin Medical University, with the approval from its Ethics Committee (approval number:KY2023-042). Lung adenocarcinoma tissues and adjacent normal lung tissues used in the experiment were obtained after surgical resection and stored in liquid nitrogen for subsequent protein extraction and western blot. In brief, then, the cells tissues were washed three times with pre-chilled PBS buffer. Total cellular proteins were extracted using RIPA lysis buffer (Beyotime Biotechnology, Shanghai, China) supplemented with a protease inhibitor cocktail (Beyotime Biotechnology, Shanghai, China) and PMSF (Beyotime Biotechnology, Shanghai, China). The BCA protein assay kit (Thermo Fisher Scientific; CA, USA) was used to determine the protein concentration. Appropriate SDS-PAGE loading buffer was added to prepare protein samples for Western blot analysis. With a protein ladder (Thermo Fisher Scientific; CA, USA) serving as the molecular weight marker, the samples were resolved by 10% SDS-PAGE gel electrophoresis. After electrophoresis, the proteins were transferred onto a PVDF membrane (Millipore, USA) for subsequent antibody incubation. We used anti- HSP90(Proteintech, Wuhan, China, 1:2000) and anti-actin (Proteintech, Wuhan, China, 1:10000) as the primary antibody, and the secondary antibody used was Goat Anti-Rabbit HRP and Goat Anti-Mouse (Proteintech, Wuhan, China, 1:10000) in this study. Finally, the visualization of protein bands was achieved using the ECL detection kit (Beyotime Biotechnology, Shanghai, China).

### Statistic analysis

R software (v. 4.2.1; https://www.r-project.org/) and its corresponding packages were used for statistical analysis and graph construction. A *p* < 0.05 was considered statistically significant. Wilcoxon rank-sum test was used to analyze differences in continuous variables between the two subgroups. Log-rank test was used to determine the contrast of K-M curves between the two groups.

### Ethical approval

All experiments were approved by the Research Ethical Committee of the second affiliated hospital of Harbin Medical University, China (approval number:KY2023-042).

### Supplementary Information

Supplementary Figures.

Supplementary Figure S1.

## Data Availability

Gene expression data for lung adenocarcinoma are available for download from the TCGA database (https://portal.gdc.cancer.gov/) and GEO database (https://www.ncbi.nlm.nih.gov/geo/). Gene sets used in this study was downloaded from the MsigDB database (http://www.gsea-msigdb.org). The scRNAseq data were retrived from Code Ocean(https://codeocean.com/;10.24433/CO.0121060.v1.).All data are available for download on the corresponding website.The western blot primary data are available in supply materials.

## References

[1] Siegel RL, Miller KD, Fuchs HE, Jemal A (2022). Cancer statistics, 2022. CA: Cancer J. Clin..

[2] Travis WD, Brambilla E, Nicholson AG, Yatabe Y, Austin JHM (2015). The 2015 World Health Organization classification of lung tumors: Impact of genetic, clinical and radiologic advances since the 2004 classification. J. Thorac. Oncol.: Off. Publ. Int. Assoc. Study Lung Cancer.

[3] Duma N, Santana-Davila R, Molina JR (2019). Non-small cell lung cancer: Epidemiology, screening, diagnosis, and treatment. Mayo Clin. Proc..

[4] Girard N, Perol M, Simon G, Audigier Valette C, Gervais R (2021). Treatment strategies for unresectable locally advanced non-small cell lung cancer in the real-life ESME cohort. Lung cancer (Amsterdam, Netherlands).

[5] Jin MZ, Jin WL (2020). The updated landscape of tumor microenvironment and drug repurposing. Signal Transduct. Target. Ther..

[6] Galluzzi L, Buqué A, Kepp O, Zitvogel L, Kroemer G (2017). Immunogenic cell death in cancer and infectious disease. Nat. Rev. Immunol..

[7] Galluzzi L, Vitale I, Warren S, Adjemian S, Agostinis P (2020). Consensus guidelines for the definition, detection and interpretation of immunogenic cell death. J. Immunother. Cancer.

[8] Zhou F, Feng B, Yu H, Wang D, Wang T (2019). Tumor microenvironment-activatable prodrug vesicles for nanoenabled cancer chemoimmunotherapy combining immunogenic cell death induction and CD47 blockade. Adv. Mater. (Deerfield Beach, Fla.).

[9] Tu K, Deng H, Kong L, Wang Y, Yang T (2020). Reshaping tumor immune microenvironment through acidity-responsive nanoparticles featured with CRISPR/Cas9-mediated programmed death-ligand 1 attenuation and chemotherapeutics-induced immunogenic cell death. ACS Appl. Mater. Interfaces..

[10] Obeid M, Tesniere A, Ghiringhelli F, Fimia GM, Apetoh L (2007). Calreticulin exposure dictates the immunogenicity of cancer cell death. Nat. Med..

[11] Massé D, Ebstein F, Bougras G, Harb J, Meflah K (2004). Increased expression of inducible HSP70 in apoptotic cells is correlated with their efficacy for antitumor vaccine therapy. Int. J. Cancer.

[12] Apetoh L, Ghiringhelli F, Tesniere A, Obeid M, Ortiz C (2007). Toll-like receptor 4-dependent contribution of the immune system to anticancer chemotherapy and radiotherapy. Nat. Med..

[13] Lazear HM, Schoggins JW, Diamond MS (2019). Shared and distinct functions of type I and type III interferons. Immunity.

[14] Mantovani A, Dinarello CA, Molgora M, Garlanda C (2019). Interleukin-1 and related cytokines in the regulation of inflammation and immunity. Immunity.

[15] Krysko DV, Agostinis P, Krysko O, Garg AD, Bachert C (2011). Emerging role of damage-associated molecular patterns derived from mitochondria in inflammation. Trends Immunol..

[16] De Munck J, Binks A, McNeish IA, Aerts JL (2017). Oncolytic virus-induced cell death and immunity: A match made in heaven?. J. Leukoc. Biol..

[17] Uprety D, Adjei AA (2020). KRAS: From undruggable to a druggable cancer target. Cancer Treat Rev..

[18] Yang W, Zhang F, Deng H, Lin L, Wang S (2020). Smart nanovesicle-mediated immunogenic cell death through tumor microenvironment modulation for effective photodynamic immunotherapy. ACS Nano.

[19] Li Y, Zhang H, Li Q, Zou P, Huang X (2020). CDK12/13 inhibition induces immunogenic cell death and enhances anti-PD-1 anticancer activity in breast cancer. Cancer Lett..

[20] Duan X, Chan C, Lin W (2019). Nanoparticle-mediated immunogenic cell death enables and potentiates cancer immunotherapy. Angew. Chem. (International ed. in English).

[21] Kroemer G, Galluzzi L, Kepp O, Zitvogel L (2013). Immunogenic cell death in cancer therapy. Annu. Rev. Immunol..

[22] Garg AD, De Ruysscher D, Agostinis P (2016). Immunological metagene signatures derived from immunogenic cancer cell death associate with improved survival of patients with lung, breast or ovarian malignancies: A large-scale meta-analysis. Oncoimmunology.

[23] Serrano-Del Valle A, Anel A, Naval J, Marzo I (2019). Immunogenic cell death and immunotherapy of multiple Myeloma. Front. Cell Dev. Biol..

[24] Whitley MJ, Suwanpradid J, Lai C, Jiang SW, Cook JL (2021). ENTPD1 (CD39) expression inhibits UVR-induced DNA damage repair through purinergic signaling and is associated with metastasis in human cutaneous squamous cell carcinoma. J. Invest. Dermatol..

[25] Xiao X, Wang W, Li Y, Yang D, Li X (2018). HSP90AA1-mediated autophagy promotes drug resistance in osteosarcoma. J. Exp. Clin. Cancer Res.: CR.

[26] Chen K, Kolls JK (2017). Interluekin-17A (IL17A). Gene.

[27] Borges da Silva H, Beura LK, Wang H, Hanse EA, Gore R (2018). The purinergic receptor P2RX7 directs metabolic fitness of long-lived memory CD8 T cells. Nature.

[28] Canaud G, Hammill AM, Adams D, Vikkula M, Keppler-Noreuil KM (2021). A review of mechanisms of disease across PIK3CA-related disorders with vascular manifestations. Orphanet J. Rare Dis..

[29] Goswami S, Walle T, Cornish AE, Basu S, Anandhan S (2020). Immune profiling of human tumors identifies CD73 as a combinatorial target in glioblastoma. Nat. Med..

[30] Zuehlke AD, Beebe K, Neckers L, Prince T (2015). Regulation and function of the human HSP90AA1 gene. Gene.

[31] Diboun I, Wernisch L, Orengo CA, Koltzenburg M (2006). Microarray analysis after RNA amplification can detect pronounced differences in gene expression using limma. BMC Genom..

[32] Seiler M, Huang CC, Szalma S, Bhanot G (2010). ConsensusCluster: A software tool for unsupervised cluster discovery in numerical data. OMICS.

[33] Tian X, Liu B, Chen L, Xie Y, Liang J (2021). RNA-seq identifies marked Th17 cell activation and altered CFTR expression in different atopic dermatitis subtypes in chinese han populations. Front. Immunol..

[34] The Gene Ontology Consortium. Expansion of the Gene Ontology knowledgebase and resources. *Nucleic Acids Research* **45**, D331-D338 (2017) 10.1093/nar/gkw1108 PMC5210579 27899567

[35] Chen H, Zhang Y, Awasthi SK, Liu T, Zhang Z (2020). Effect of red kaolin on the diversity of functional genes based on Kyoto encyclopedia of genes and genomes pathways during chicken manure composting. Biores. Technol..

[36] Kanehisa M (2019). Toward understanding the origin and evolution of cellular organisms. Protein Sci.: Publ. Protein Soc..

[37] Kanehisa M, Furumichi M, Sato Y, Kawashima M, Ishiguro-Watanabe M (2023). KEGG for taxonomy-based analysis of pathways and genomes. Nucleic Acids Res..

[38] Kanehisa M, Goto S (2000). KEGG: kyoto encyclopedia of genes and genomes. Nucleic Acids Res..

[39] Subramanian A, Tamayo P, Mootha VK, Mukherjee S, Ebert BL (2005). Gene set enrichment analysis: A knowledge-based approach for interpreting genome-wide expression profiles. Proc. Natl. Acad. Sci. U.S.A..

[40] Kim N, He N, Kim C, Zhang F, Lu Y (2012). Systematic analysis of genotype-specific drug responses in cancer. Int. J. Cancer.

[41] Mayakonda A, Lin DC, Assenov Y, Plass C, Koeffler HP (2018). Maftools: efficient and comprehensive analysis of somatic variants in cancer. Genome Res..

[42] Yoshihara K, Shahmoradgoli M, Martínez E, Vegesna R, Kim H (2013). Inferring tumour purity and stromal and immune cell admixture from expression data. Nat. Commun..

[43] Jiang P, Gu S, Pan D, Fu J, Sahu A (2018). Signatures of T cell dysfunction and exclusion predict cancer immunotherapy response. Nat. Med..

[44] Macosko EZ, Basu A, Satija R, Nemesh J, Shekhar K (2015). Highly Parallel Genome-wide Expression Profiling of Individual Cells Using Nanoliter Droplets. Cell.

