# Supplementary figures and images for: Immunogenic cell death-based prognostic model for predicting the response to immunotherapy and common therapy in lung adenocarcinoma
Source: Sci Rep. 2023 Aug 16;13:13305. doi: 10.1038/s41598-023-40592-w (PMC10432465; doi:10.1038/s41598-023-40592-w)

**R1:**

HSP90:

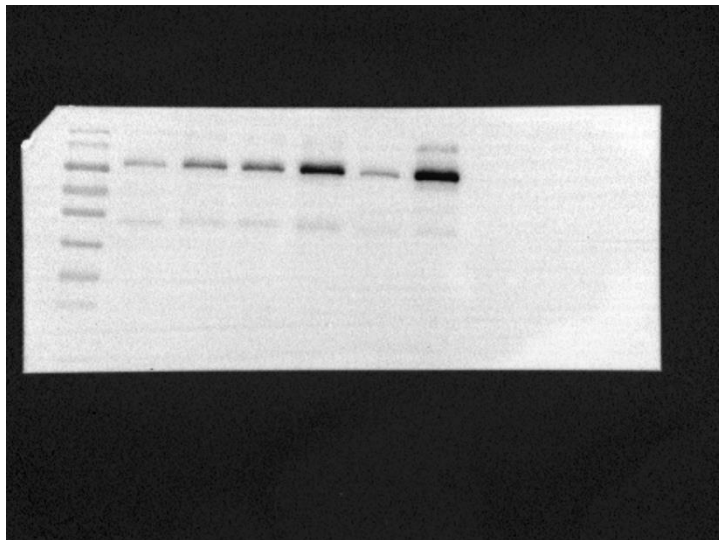

$\beta$ -actin:

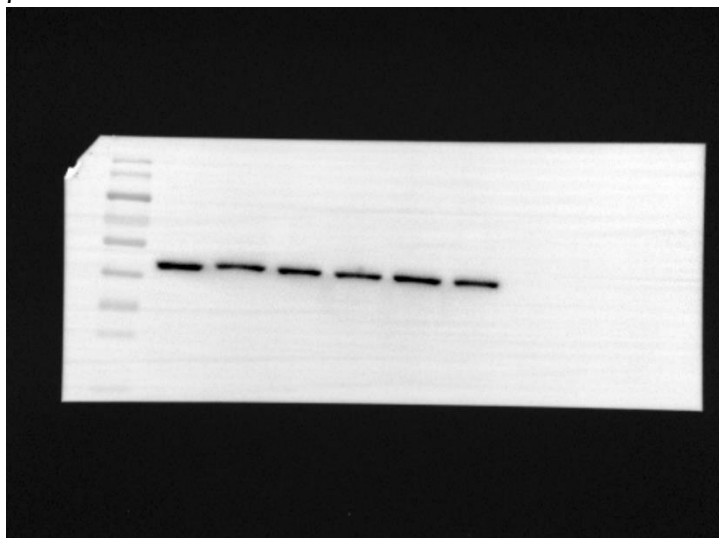

**R2:**

HSP90:

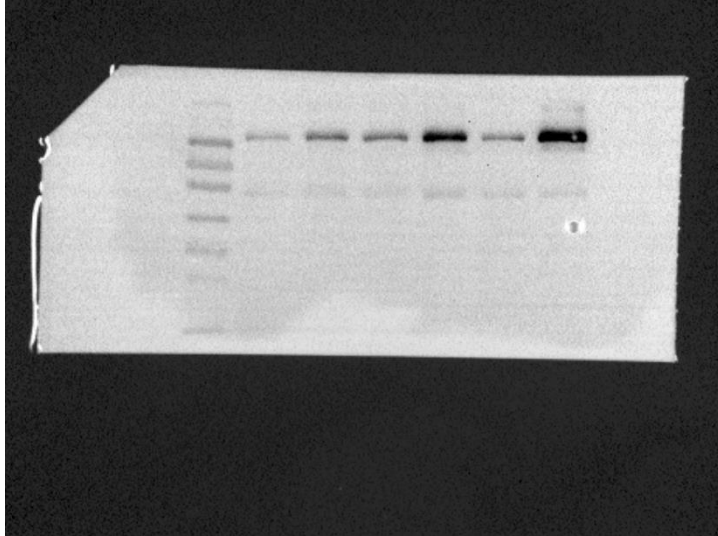

$\beta$ -actin:

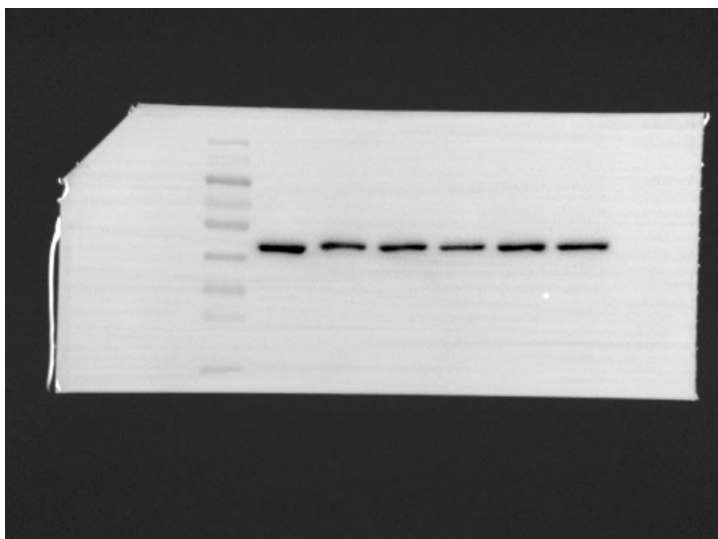

**R3:**

HSP90:

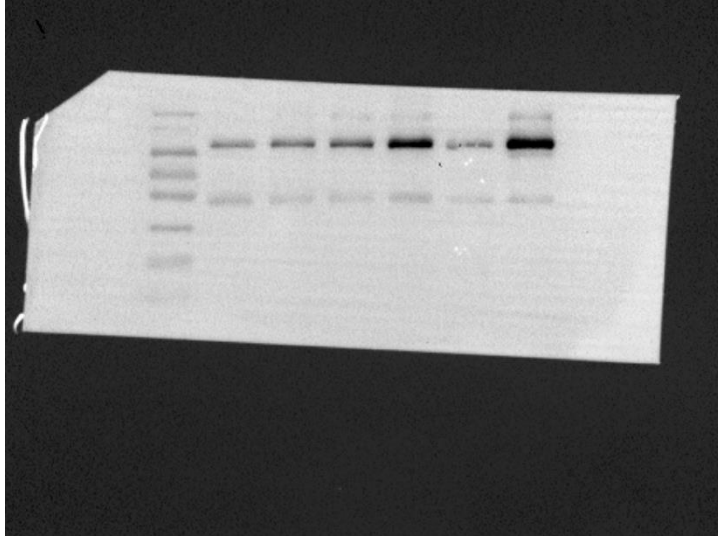

$\beta$ -actin:

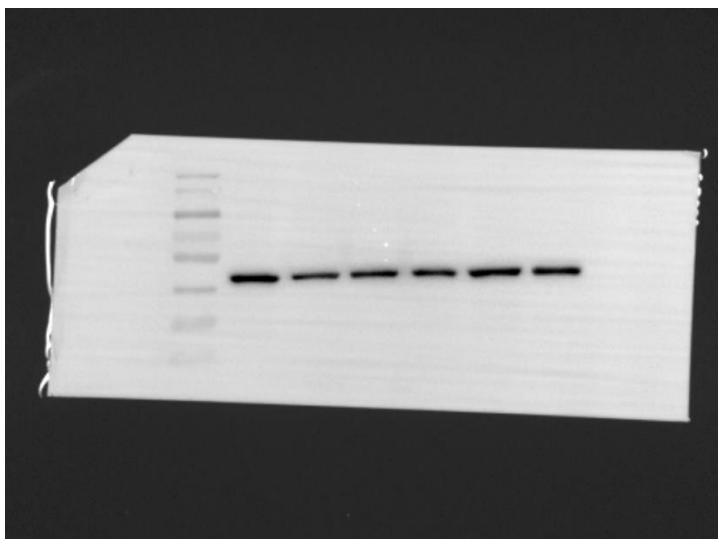

Supplement: Supplementary file 1 — Supplementary Figures. [file 41598_2023_40592_MOESM1_ESM.pdf]
