# Supplementary material for: Immunogenic cell death-based prognostic model for predicting the response to immunotherapy and common therapy in lung adenocarcinoma
Source: Sci Rep. 2023 Aug 16;13:13305. doi: 10.1038/s41598-023-40592-w (PMC10432465; doi:10.1038/s41598-023-40592-w)
Supplement: Supplementary file 2 — Supplementary Figure S1. [file 41598_2023_40592_MOESM2_ESM.docx]

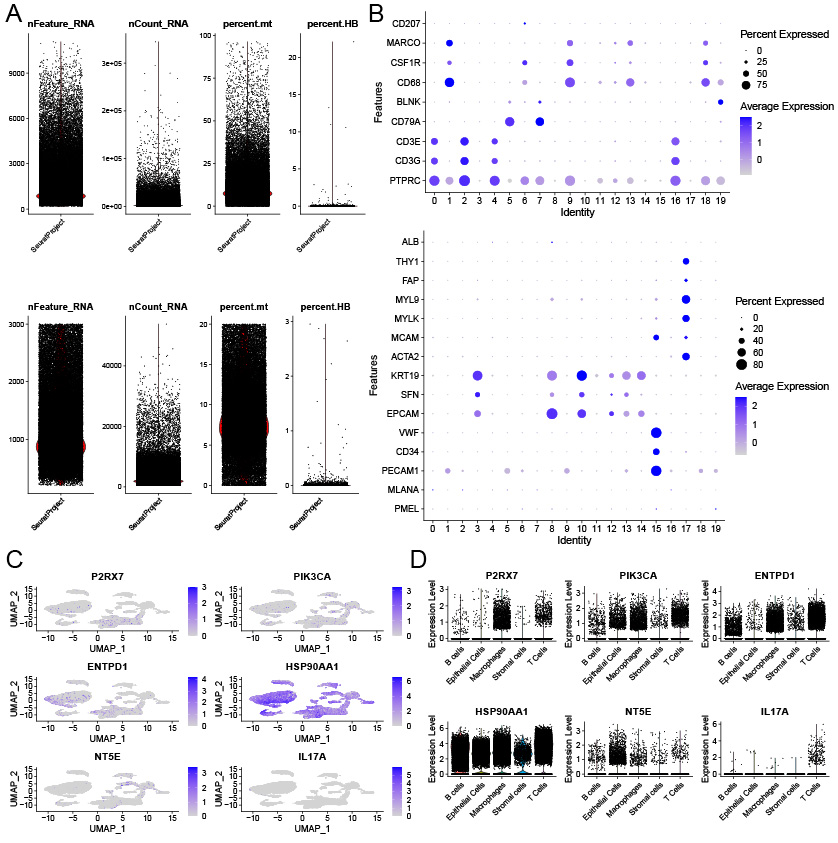


**Figure S1:**scRNAseq analysis.(A)Vinplot before and after quality control process. (B)Dotplot of immune and other cell biomarker expressions in 20 clusters.(C)Feature plot of 6 ICD-related genes used for model construction. (D)Vinplot of 6 ICD-related genes used for model construction.
